# Genome-wide evolution and function analysis of ALOG gene family in cotton

**DOI:** 10.3389/fgene.2025.1625634

**Published:** 2025-09-10

**Authors:** Zhen Liu, Siyu Shen, Zhijuan Cui, Tao Wang, Pengtao Li, Yangyang Wei, Renhai Peng

**Affiliations:** ^1^ Anyang Key Laboratory of Bioinformatics, School of Biotechnology and Food Science, Anyang Institute of Technology, Anyang, Henan, China; ^2^ School of Computer and Artificial Intelligence, Zhengzhou University, Zhengzhou, Henan, China; ^3^ Tangyin County Agriculture and Rural Bureau, Tangyin, Henan, China

**Keywords:** cotton, ALOG, development, evolution, function

## Abstract

**Background:**

The ALOG (*Arabidopsis thaliana LSH1* and *Oryza sativa G1*) gene family is a class of transcription factors present in various plants. To elucidate the roles of ALOG genes in cotton, we systematically investigated the ALOG gene family across four cotton species (*Gossypium hirsutum*, *Gossypium barbadense*, *Gossypium arboreum* and *Gossypium raimondii*).

**Results:**

In this study, a total of 43, 42, 23 and 27 ALOG genes were identified from *G. hirsutum*, *G. barbadense*, *G. arboretum* and *G. raimondii*, respectively. The results indicated that cotton ALOG gene duplications originated before the speciation of *Gossypium* species, whole genome duplication, segmental duplication and transposable elements all play important roles in its expansion. In addition, cotton ALOG genes had undergone purifying selection during the evolution. Cis-element analysis revealed that TATA-box and CAAT-box are the most abundant in the promoters of cotton ALOG genes. Transcriptome analysis showed that the expression of ALOG genes in specific tissue is significantly higher than that in other tissues.

**Conclusion:**

This study enhances our comprehension of cotton ALOG genes, and these findings lay the foundation for functional characterizations of ALOG gene family.

## Introduction

The Arabidopsis *LSH1* and Oryza *G1* (ALOG) gene family is a plant-specific transcription factor (Iyer and Aravind, 2012; Li et al., 2019). The N-terminus of ALOG family proteins fused with a N6-adenine methylase active region, and the C-terminus fused with a tyrosine recombinase catalytic active region (Iyer and Aravind, 2012). Studies indicated that the ALOG gene family plays regulatory roles in various aspects of plant growth and development in different lineages of land plants. For example, in rice, there is evidence to suggest that *OsG1L1* and *OsG1L2* have significant effects on inflorescence development (Beretta et al., 2023). In *Arabidopsis thaliana*, the ALOG genes of *LSH4* and *LSH3* are known to suppress organ differentiation the boundary region of the shoot apical meristem (Rieu et al., 2024). In *Torenia fournieri*, *TfALOG3* is associated with corolla tube development and differentiation, and the expression level of *TfALOG3* gene is significantly high in corolla tube. Cells in the corolla bottom differentiated and expanded in wild-type *Torenia fournieri*, whereas such cells in TfALOG3 loss-of-function mutants failed to develop into a corolla neck (Xiao et al., 2019; Xiao et al., 2018).

Cotton belongs to the Malvaceae family and the *Gossypium* genus, with more than fifty species. The diploid cotton genome is grouped into eight groups, designated A-K, allotetraploid species, such as *G. hirsutum* and *G. barbadense*, originated from the hybridization of A and D genomes (Grover et al., 2012). Cotton fiber is a critical source of fiber for the textile sector. The development of cotton fibers begins with a single cell protrusion on the ovule epidermis, and then differentiates into elongated and thickened seed trichome (Zhai et al., 2023). Although the ALOG gene family plays an important role in plant growth and development, little is known about its molecular mechanism in cotton fiber development; therefore, it would be interesting to make a systematic investigation of the ALOG family in cotton plants. In this study, we carried out a whole-genome identification and analysis of cotton ALOG gene family, including their phylogenetic relationships, conserved motif, selection pressure, evolution, cis-elements and function. Our study will provide a foundation for downstream functional investigation of ALOG genes, and will provide insights into the understanding of the regulatory mechanisms of ALOG genes in controlling fiber growth.

## Materials and methods

### Identification of cotton ALOG gene family

Genome sequences and annotation files of *G. hirsutism* (Wang et al., 2019), *G. barbadense* (Wang et al., 2019), *G. arboretum* (Du et al., 2018) and *G. raimondii* (Udall et al., 2019) were downloaded from COTTONGENE (http://www.cottongen.org). The hidden Markov model of ALOG (PF04852) were obtained from the InterPro database (https://www.ebi.ac.uk/interpro/), which were used to retrieve cotton ALOG proteins by HMMER (Finn et al., 2011). In addition, we performed a sequence similarity search by BLAST (Matsuda et al., 2013; Nowicki et al., 2018) (E value ≤ E^−10^) with the ALOG amino acid sequences of *Arabidopsis thaliana* and *Oryza sativa* as queries (Li et al., 2019). Then HMMER results were combined with the BLAST search results, and NCBI-CDD-Search (Yang et al., 2020) was used for further confirmation. The physical and chemical properties of cotton ALOG proteins were predicted by the software Compute pI/Mw (https://web.expasy.org/compute_pi/).

### Phylogenetic and conserved motif analysis of cotton ALOG gene family

Multiple sequence alignment of ALOG proteins was carried out by the Clustal X (Larkin et al., 2007). A maximum likelihood tree with a bootstrap value of 1,000 was constructed by MEGA (Hall, 2013). The result tree was then decorated by iTOL (https://itol.embl.de/upload.cgi) (Letunic and Bork, 2021). The online tool MEME (Bailey et al., 2009) (https://meme-suite.org/meme/) was used to analysis conserved motifs, with motif number set to 5. The cis-elements in promoter sequences upstream 1,500 bp of ALOG genes were predicted by PlantCARE (Lescot et al., 2002). The exon-intron organization of cotton ALOG genes was identified by GSDS (Hu et al., 2015) (https://gsds.gao-lab.org/).

### Gene duplication and synteny analyses of cotton ALOG gene family

The collinearity relationships of ALOG genes were analyzed by MCScanX (Wang et al., 2012), and the results were visualized using Circos (Krzywinski et al., 2009). Ka/Ks ratios between ALOG members was calculated by KaKs_Calculator software (Wang et al., 2010). The divergence time was calculated by the formula T = Ks/2λ, where λ represents the neutral substitution rate, which is set to 1.5 × 10^−8^ in this study (Koch et al., 2000).

### Transposable elements analysis of cotton ALOG gene family

The transposable elements library was construct by RepeatModeler, and RepeatMasker was used to predict transposable elements (Tarailo-Graovac and Chen, 2009; Tempel, 2012). The transposable elements in 2,000 bp and 10,000 bp upstream and downstream regions of the ALOG genes were identified in this study.

### Expression profile analysis of cotton ALOG gene family

Transcriptome data were downloaded from NCBI SRA database (https://www.ncbi.nlm.nih.gov/sra/). The SRA data for multiple tissues (PRJNA490626), different fiber development stages (PRJNA263926) and long day and short day conditions (PRJNA529417) were converted to fastq format with the SRA Toolkit. The software Trimmomatic (Bolger et al., 2014) was used to remove the adapters and to perform quality control and hisat2 (Kim et al., 2015) was used to map the reads to the genomes. Transcript abundance for ALOG genes was quantified using the fragments per kilobase million (FPKM) metric, which was calculated by Cuffinks software (Ghosh and Chan, 2016). Heatmaps of the expression profile values were generated with pheatmap package of R language.

### Protein interaction network analysis of cotton ALOG gene family

The interaction network of ALOG proteins was predicted by the online website STRING (https://string-db.org/) (Szklarczyk et al., 2015). *Arabidopsis thaliana* was selected as the organism to retrieve the protein interaction network map.

## Result

### Identification of cotton ALOG gene family

In the present study, a total of 43, 42, 23 and 27 ALOG genes were identified from *G. hirsutum*, *G. barbadense*, *G. arboretum* and *G. raimondii*, respectively (Supplementary Table S1). The numbers of ALOG genes in diploid cotton (*G. arboretum* and *G. raimondii*) was found to be comparable to that of *Nicotiana* species (12–23) (Turchetto et al., 2023), yet it exceeded the number observed in *Arabidopsis* (10) (Rieu et al., 2024).

In addition, the results showed that the numbers of ALOG genes in diploid cotton species are almost half those of tetraploid cotton species. Therefore, we speculate that the ALOG gene family likely originated in diploid cotton species and expanded during polyploidization. However, some gene losses may have occurred post-polyploidization.

To further characterize the cotton ALOG proteins, molecular weight, amino acid sequence length and isoelectric point value were analyzed. Notably, the sequence of Ghir_D10G021590.1 in *G. hirsutum*, Gbar_D10G021160.1 in *G. barbadense* and Ga10G0697 in *G. arboretum* are significantly longer than other ALOG proteins. The NCBI-CDD database revealed that these three proteins contain not only the ALOG domain but also a LRR domain (NCBI CDD: 443914), while all other proteins only contain the ALOG domain. Proteins containing LRR domain include tyrosine kinase receptors, cell-adhesion molecules, virulence factors, and extracellular matrix-binding glycoproteins, and are involved in a variety of biological processes, including signal transduction, cell adhesion, DNA repair, recombination, transcription, RNA processing, disease resistance, apoptosis, and the immune response (Kobe and Kajava, 2001).

Except for these 3 special ALOG proteins, the sequence length of cotton ALOG proteins range from 142 aa (Ghir_A07G015930.1) to 280 aa (Ga08G2374), and the isoelectric point ranged from 8.36 (Ghir_D13G025500.1) to 10.41 (Ghir_D02G004400.1). According to the results, cotton ALOG proteins have a wide range of sequence length and isoelectric point, however, the statistical results of them in the 4 cotton species are very similar, for example, the average molecular weight is around 23,627 Da, the average number of amino acids is about 215 aa, and the average isoelectric point is around 9.7.

### Phylogenetic analyses of cotton ALOG gene family

**FIGURE 1:**
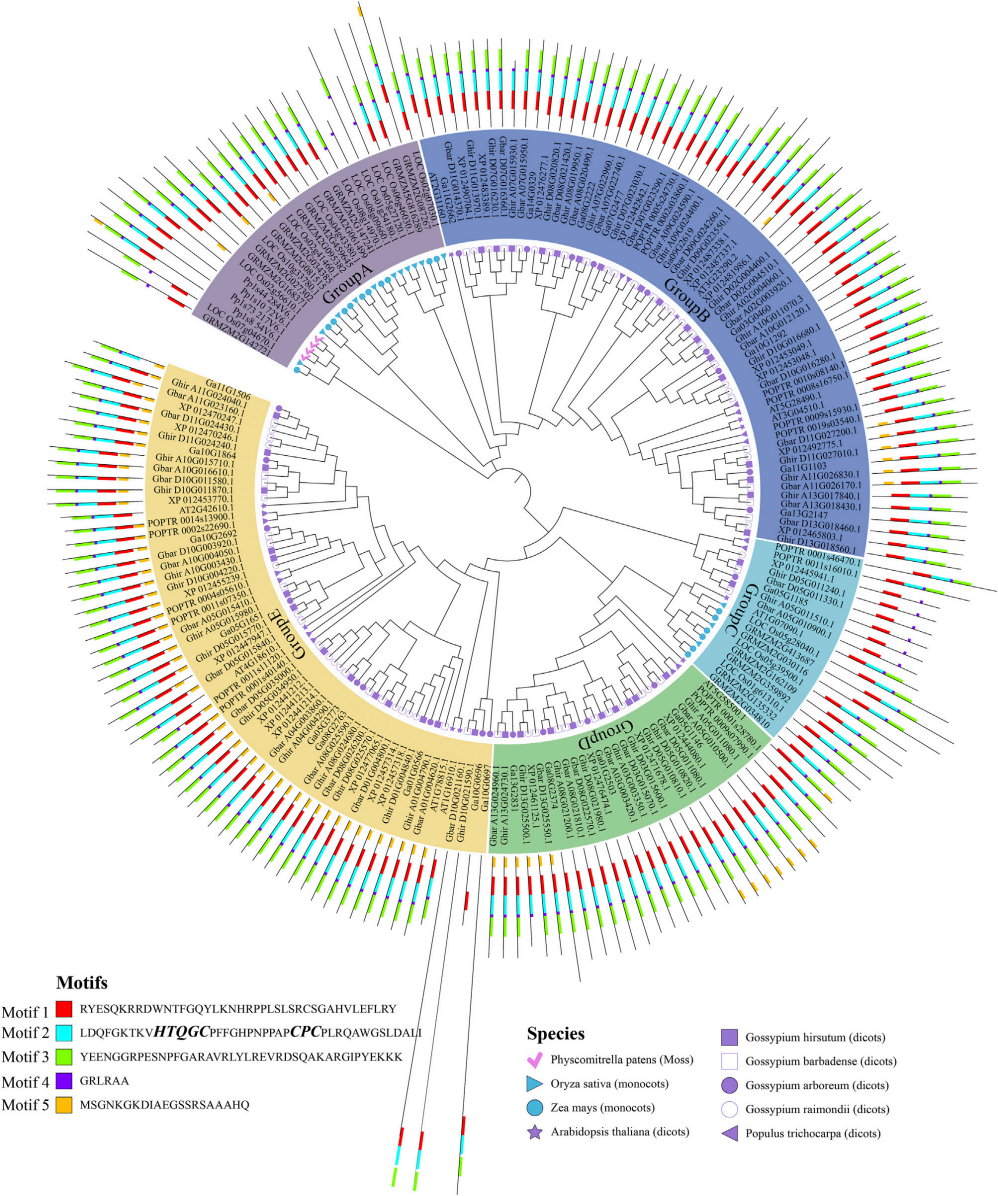
Phylogenetic tree and conserved motif analysis of the ALOG gene family.

To better understand the origin and diversification of the ALOG gene family, a phylogenetic tree was inferred with ALOG protein sequences of *Physcomitrella patens*, *Oryza sativa*, *Zea mays*, *Arabidopsis thaliana, Populus trichocarpa* and the 4 cotton species. The ALOG proteins can be divided into five groups (Figure 1), among them, the group B and E contain a larger number of proteins. Noticeably, most *Physcomitrella patens*, *Oryza sativa* and *Zea mays* ALOG proteins were distributed in group A and C, while *Arabidopsis thaliana* and *Populus trichocarpa* ALOG proteins were distributed in group B, D and E along with 4 cotton species. This result indicated that ALOG proteins from the same monocot species are clustered into a branch, but those from the same dicot species are dispersed into different branches. Furthermore, Figure 1 shows that many sub-groups contain similar numbers of ALOG family from the 4 cotton species and other dicot species, which suggested that the expansion has occurred before the divergence of dicot species.

### Gene structure and conserved motif of cotton ALOG gene family

A total of 5 motifs were identified from the conserved domains of cotton ALOG proteins. The majority members of ALOG proteins contain motif 1–4, indicating that they are the main motifs that make up the ALOG domain (Figure 1). Motif 5 were mainly present in the group E. Group C could be further divided into 2 sub-groups. One of the sub-groups contain *Oryza sativa* and *Zea mays*ALOG proteins, and all of them contain motif 1–4, which is similar to the majority members of ALOG proteins. More interestingly, the other sub-group included 1 ALOG proteins from each diploid cotton species and 2 ALOG proteins from each tetraploid cotton species, and all of them only contain motif 1 and motif 4. These results suggested that ALOG members of group C originated very early in evolution, and they were very conserved since the divergence of cotton species.

Studies have shown that the ALOG domain includes two conserved regions: N-terminal DNA-binding region and C-terminal region (Liu et al., 2024). The sequence of N-terminal with a “HxxxC” and “CxC” signature, which is consistent with Motif 2 (Rieu et al., 2024). The C-terminal region is fused to a tyrosine recombinase catalytic region (Rieu et al., 2024), which is consistent with Motif 1 (Figure 1). In contrast, the functions of Motif 3–5 remain unknown.

The exons and introns were analyzed to get better understand the gene structural evolution of cotton ALOG gene family (Roy and Gilbert, 2006). The results showed that 80.7% (109/135) cotton ALOG genes did not contain introns (Supplementary Table S1). The majority of ALOG genes belonging to group B contain introns, and most of them contained only one intron. Additionally, in group E, 1 ALOG gene from *G. hirsutum*, *G. barbadense* and *G. arboretum* contain 3 introns. Taken together, it appears that genes from the same group also have similar motif and gene structure features, so there may be consistency in the protein function.

### Gene duplication of cotton ALOG gene family

**FIGURE 2:**
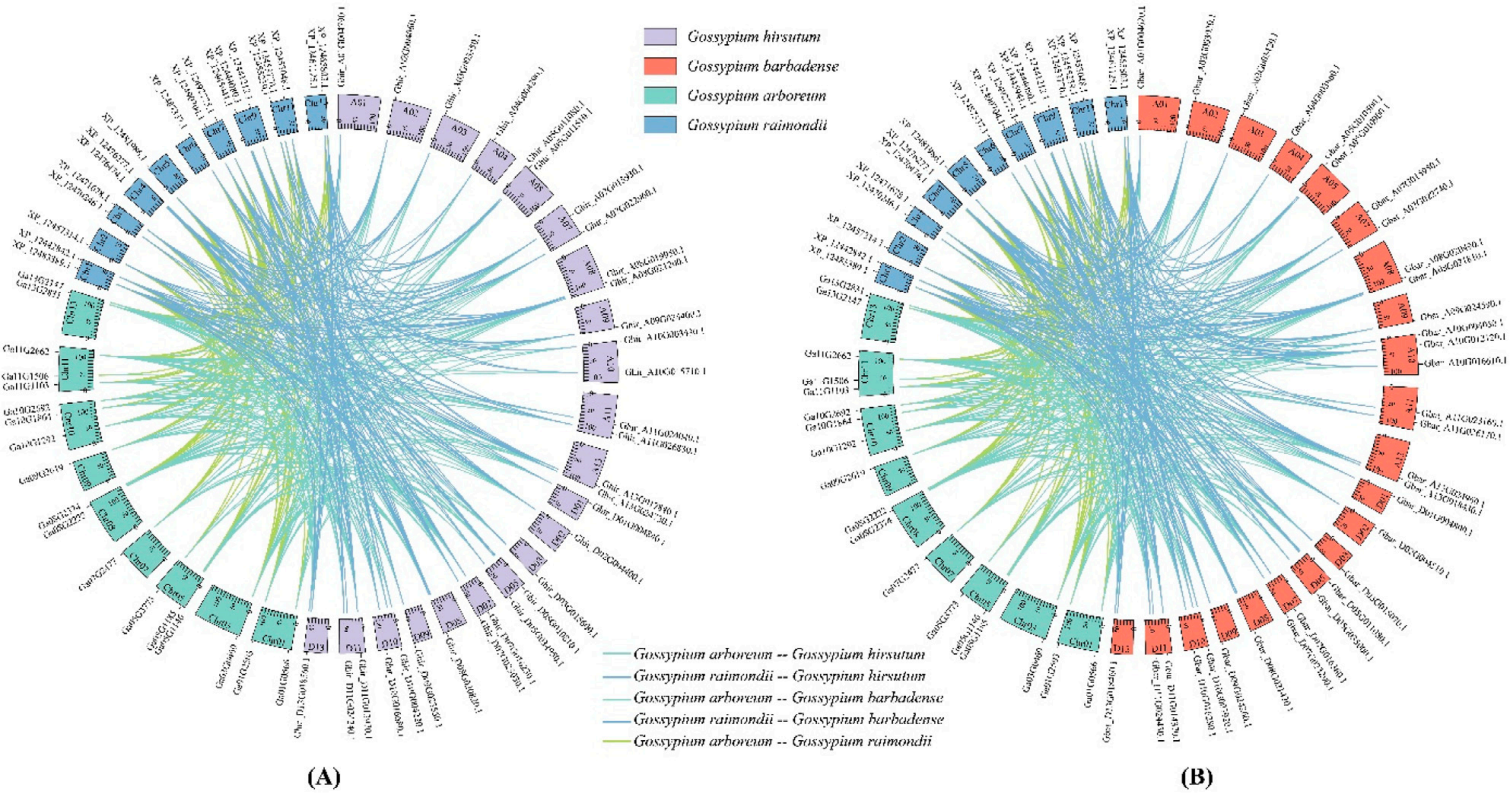
Segmental duplication relationship between cotton ALOG genes. **(A)** Allotetraploid *Gossypium hirsutum* and diploid *Gossypium arboretum*, *Gossypium raimondii*; **(B)** Allotetraploid *Gossypium barbadense* and diploid *Gossypium arboretum*, *Gossypium raimondii*.

The genome chromosomal distribution results indicated that ALOG genes were unevenly distributed on different chromosomes, and most chromosomes contain 1–2 ALOG genes (Figure 2). In addition, Ga14G0329 of *G. arboretum* were located on scaffolds. We refer to the description of Holub that two or more genes of the same family within 200 kb on the same chromosomal is a tandem duplication event (Holub, 2001). There were 2 ALOG genes (Ga10G0696, Ga10G0697) clustered into one tandem duplication event regions on *G. arboretum* chromosomes Chr10, but no tandemly duplicated genes were found in *G. hirsutum*, *G. barbadense* and *G. raimondii*.

Segmental duplicate gene pairs were searched by MCScanX. The results indicated that 40, 41, 18, and 17 genes formed 110, 140, 27, and 28 segmental duplication pairs in *G. hirsutum*, *G. barbadense*, *G. arboretum* and *G. raimondii*, respectively, accounting for 93.02%, 97.62%, 78.26%, and 62.96% of the ALOG gene family. *G. hirsutum* and *G. barbadense* (AADD) are typical allotetraploid from its diploid ancestors *G. arboreum* (AA) and *G. raimondii* (DD).

Segmental duplication relationships between the subgenome and the corresponding ancestral diploid genomes were analyzed to understand the evolutionary mechanism of cotton ALOG gene family. In *G. hirsutum*, 39 ALOG genes had orthologs in the *G. arboreum*, 40 genes had orthologs in the *G. raimondii*, while only 3 genes (Ghir_D10G021590.1, Ghir_D05G010820.1 and Ghir_A10G011070.3) had no ortholog. In *G. barbadense*, similarly, 40 ALOG genes had orthologs in the *G. arboreum*, 41 genes had orthologs in the *G. raimondii*, while only 1 gene (Gbar_D10G021160.1) had no ortholog (Figure 2).

### The selection pressure of cotton ALOG gene family

**FIGURE 3:**
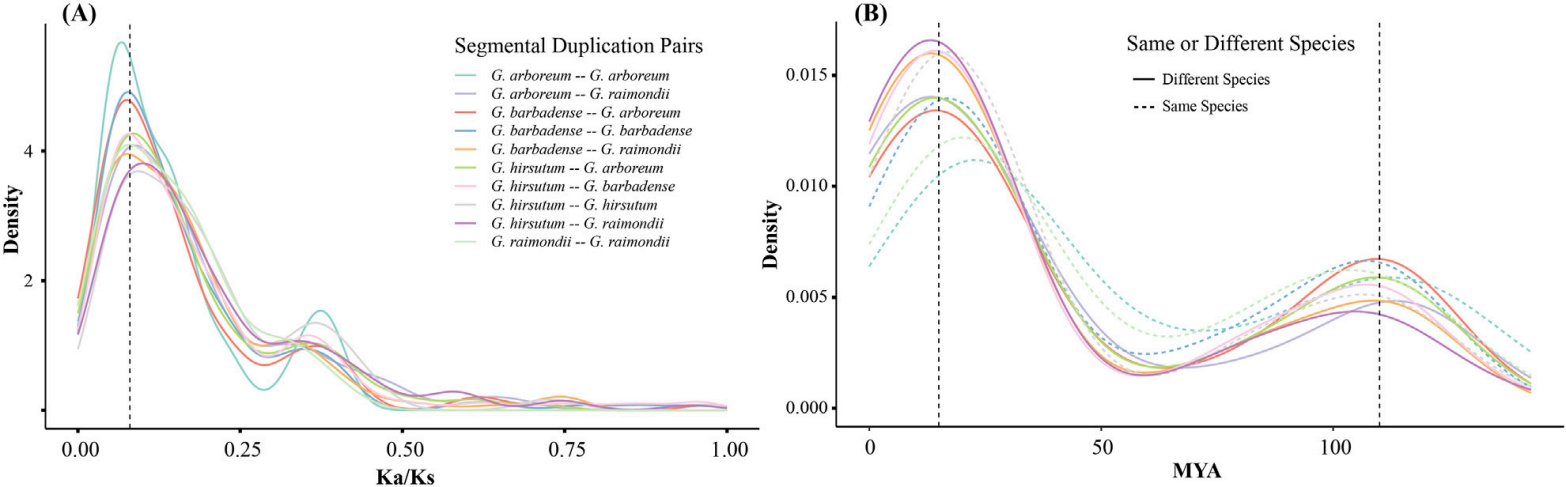
The distribution of Ka/Ks ratio and divergence time of cotton ALOG gene family. **(A)** Ka/Ks ratio distribution; **(B)** Divergence time distribution.

In order to further the understanding of the evolutionary constraints of the ALOG gene family, an analysis was conducted to determine the Ka/Ks ratio. The Ka/Ks ratios of ALOG segmental duplication gene pairs between same or different species are all around 0.08 (Figure 3A). These results suggested that cotton ALOG genes were under strong purifying selection (Hurst, 2002). Furthermore, we estimated the divergence time of cotton ALOG gene family by Ks values. The results indicate that the divergence time of ALOG segmental duplication gene pairs concentrated around 15 MYA (million years ago) and 110 MYA (Figure 3B). Previous studies have estimated that the divergence of cotton species began around 10 MYA (Chen et al., 2016; Chen et al., 2017). Based on these results, we speculate that most of the ALOG duplications took place before the speciation of cotton species. In addition, researches have shown that a major polyploidy event occurred within the eudicots around 117 MYA (Jiao et al., 2012), which suggests that the event has an important impact on the expansion of ALOG gene family.

### Transposable element analysis of cotton ALOG gene family

Transposable elements are widely distributed in the genome, especially in plants, which are important for genome expansion and evolution. We identified the transposable elements located 2,000 bp upstream and downstream of the ALOG genes. Our results indicate that 18.60% (8/43), 19.05% (8/42), 13.04% (3/23), and 25.93% (7/27) of ALOG genes close to transposable elements in *G. hirsutum*, *G. barbadense*, *G. arboretum* and *G. raimondii*, respectively. Of these transposable elements, most of them are DNA transposon. When the scanning region broadened to 10,000 bp upstream and downstream, 74.72% (32/43), 69.05% (29/42), 60.87% (14/23), and 66.67% (18/27) genes were found near the transposable elements in *G. hirsutum*, *G. barbadense*, *G. arboretum* and *G. raimondii*, respectively. In addition to DNA transposons, many LTR retrotransposons were identified (Supplementary Table S2). Therefore, our results indicate that cotton ALOG gene family has expansion due to the activity of transposable elements.

### Key cis-elements analysis of cotton ALOG gene family

**FIGURE 4:**
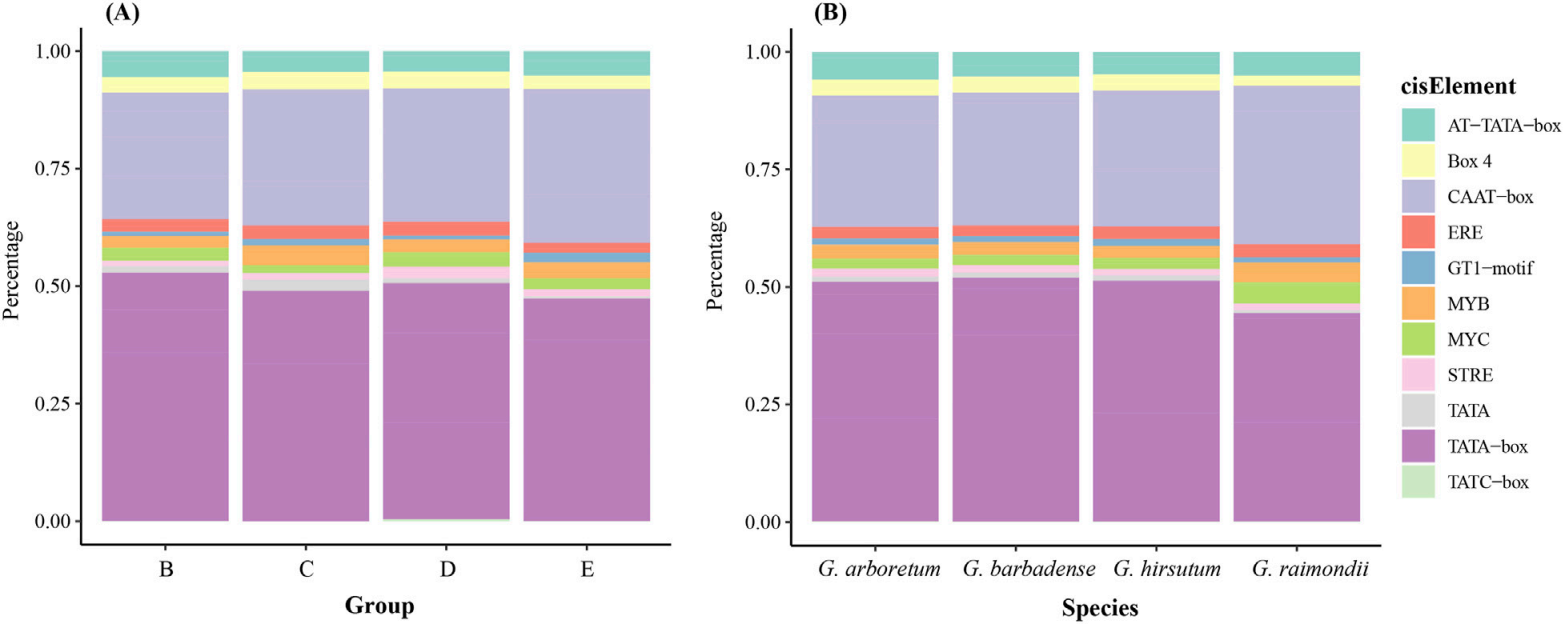
Cis-elements composition proportion of cotton ALOG gene. **(A)** Different group; **(B)** Different species.

The cis-element present in the promoters of cotton ALOG genes were identified using PlantCARE. The result indicates that TATA-box and CAAT-box were the most abundant cis-elements, in addition, there were also cis-elements related to stress responses (MYB, MYC, STRE), light responsiveness (Box 4, GT1-motif) and so on (Figure 4). Furthermore, we found that the proportions of these cis-elements are similar across different species and groups which indicated the cis-elements of ALOG genes are very conserved after the divergence of cotton species.

### Expression patterns of cotton ALOG genes in different tissues

**FIGURE 5:**
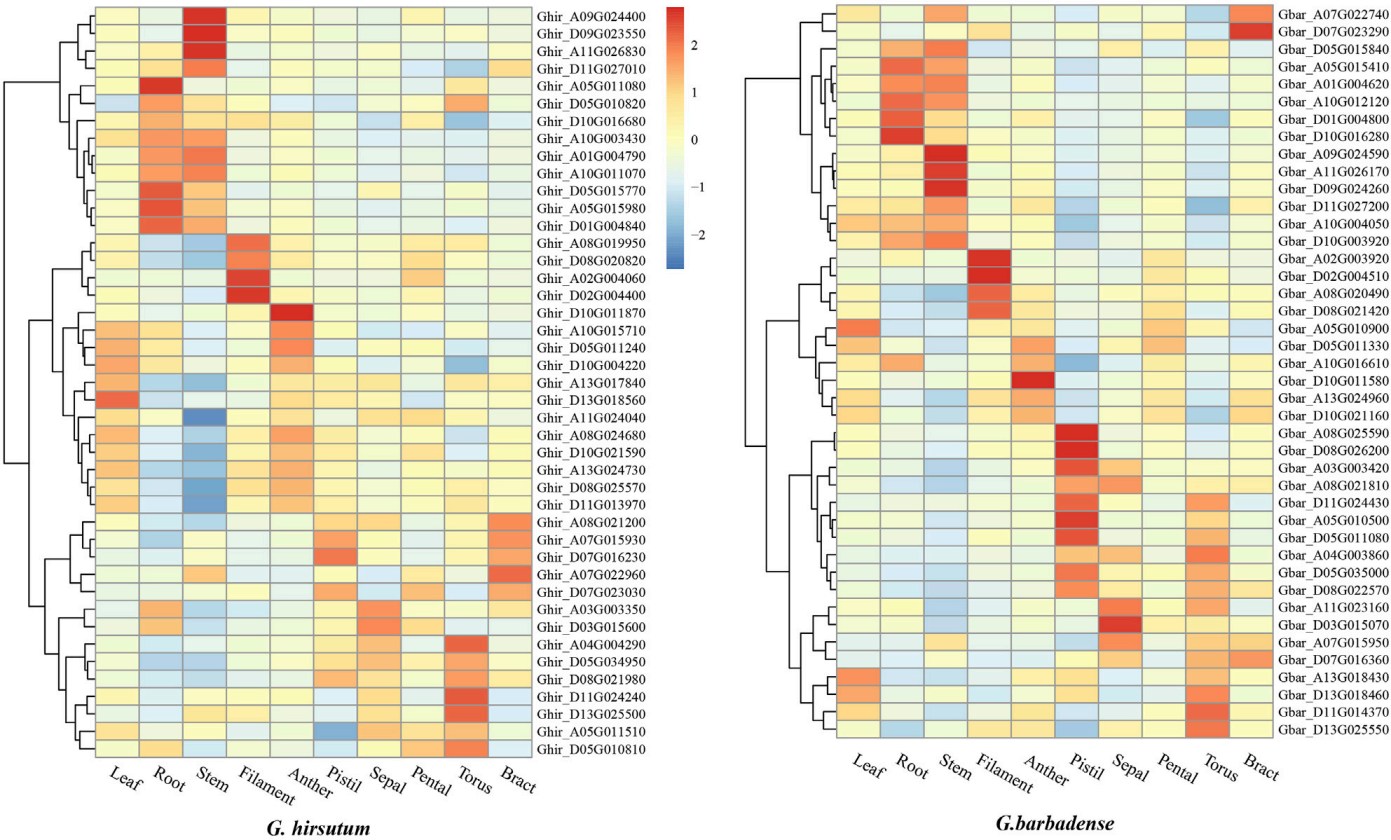
Expression heatmap of *Gossypium hirsutum* and *Gossypium barbadense* ALOG genes in different tissues.

We analyzed the transcriptomic data of leaf, root, stem, filament, anther, pistil sepal, pental, torus and bract in *G. hirsutum* and *G. barbadense*. The results indicated that most ALOGs showed tissue-specific expression patterns, As shown in Figure 5, Ghir_A09G024400, Ghir_D09G023550, Ghir_A11G026830 of *G. hirsutum* and Gbar_A09G024590, Gbar_A11G026170, Gbar_D09G024260 of *G. barbadense* exhibited significantly higher expression in stem than other tissues. Similarly, Ghir_A08G019950, Ghir_D08G020820, Ghir_A02G004060, Ghir_D02G004400 of *G. hirsutum* and Gbar_A02G003920, Gbar_D02G004510, Gbar_A08G020490, Gbar_D08G021420 of *G. barbadense* were mainly expressed in filament but barely expressed in other tissues. Therefore, it perhaps that ALOG gene mainly play its role in a specific tissue. In addition, some ALOG genes expressed in the same tissue were segmental duplicate gene pairs, for example, Ghir_A09G024400, Ghir_D09G023550, Gbar_A09G024590 and Gbar_D09G024260, but not all genes were like this, for example, Ghir_A11G026830 and Gbar_A11G026170 were both highly expressed in the stem, but they were not segmental duplicate gene pairs. Furthermore, members sharing closer phylogenetic relationships displayed similar expression patterns, for example, the six ALOG genes highly expressed in stem were all belong to Group B.

### Expression patterns of cotton ALOG genes at different fiber development stages

**FIGURE 6:**
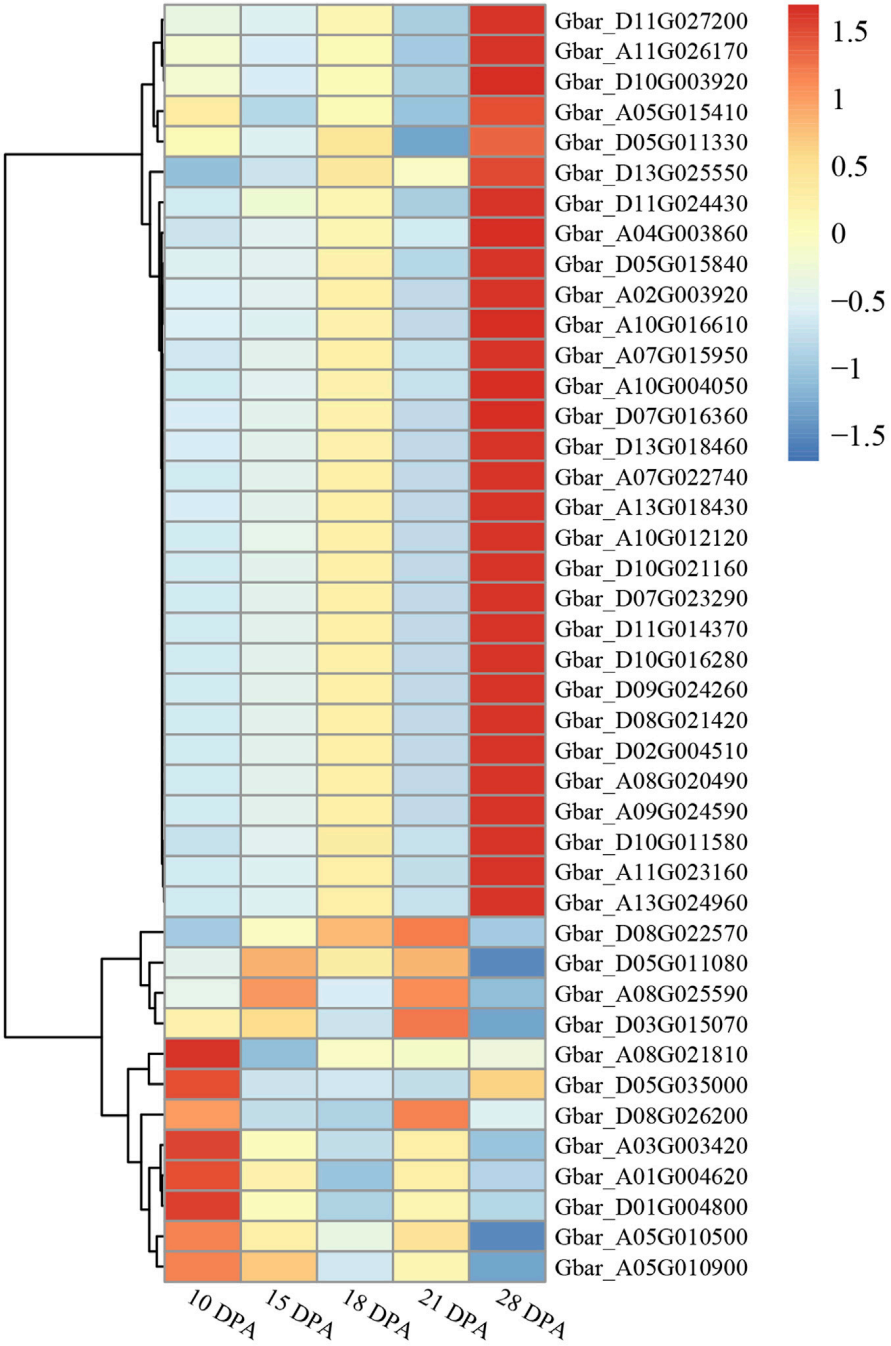
Expression profile of *Gossypium barbadense* ALOG genes at different stages of fiber development.

To explore the potential role of ALOG in fiber development, we investigated their expression at different fiber development stages (10–28 DPA) of *G. barbadense* by RNA-sequencing. The results indicated that the expression pattern of ALOG genes can be grouped into 3 clusters (Figure 6). Thirty *G. barbadense* ALOG genes showed upregulated expression during fiber development, whereas eight exhibited downregulation. In addition, the expression of Gbar_D08G022570, Gbar_D05G011080, Gbar_A08G025590, Gbar_D03G015070 exhibited differences at different stages of fiber development. Based on the above RNA-seq data analysis result, we believe that the ALOG genes play a significant role in the fiber development.

### Expression patterns of cotton ALOG genes in long day and short day conditions

To investigate the potential function of ALOG family in long day and short day conditions, the transcriptome data from leaf and mristem of *G. hirsutum*, *G. barbadense*, *G. arboretum* and *G. raimondii* was used to calculate the expression of ALOG genes. The expression ratio for the long day conditions/short day conditions was calculated for the expression pattern of each ALOG genes. In the calculation, we only keep the data with FPKM>1 in both conditions. The results showed that 64.5% of the family members had ratios less than 0.8 or more than 1.2, of which 23.5% is less than 0.8% and 41.0% is more than 1.2 (Supplementary Table S3).

### Protein interaction network and functional annotation of cotton ALOG proteins

**FIGURE 7:**
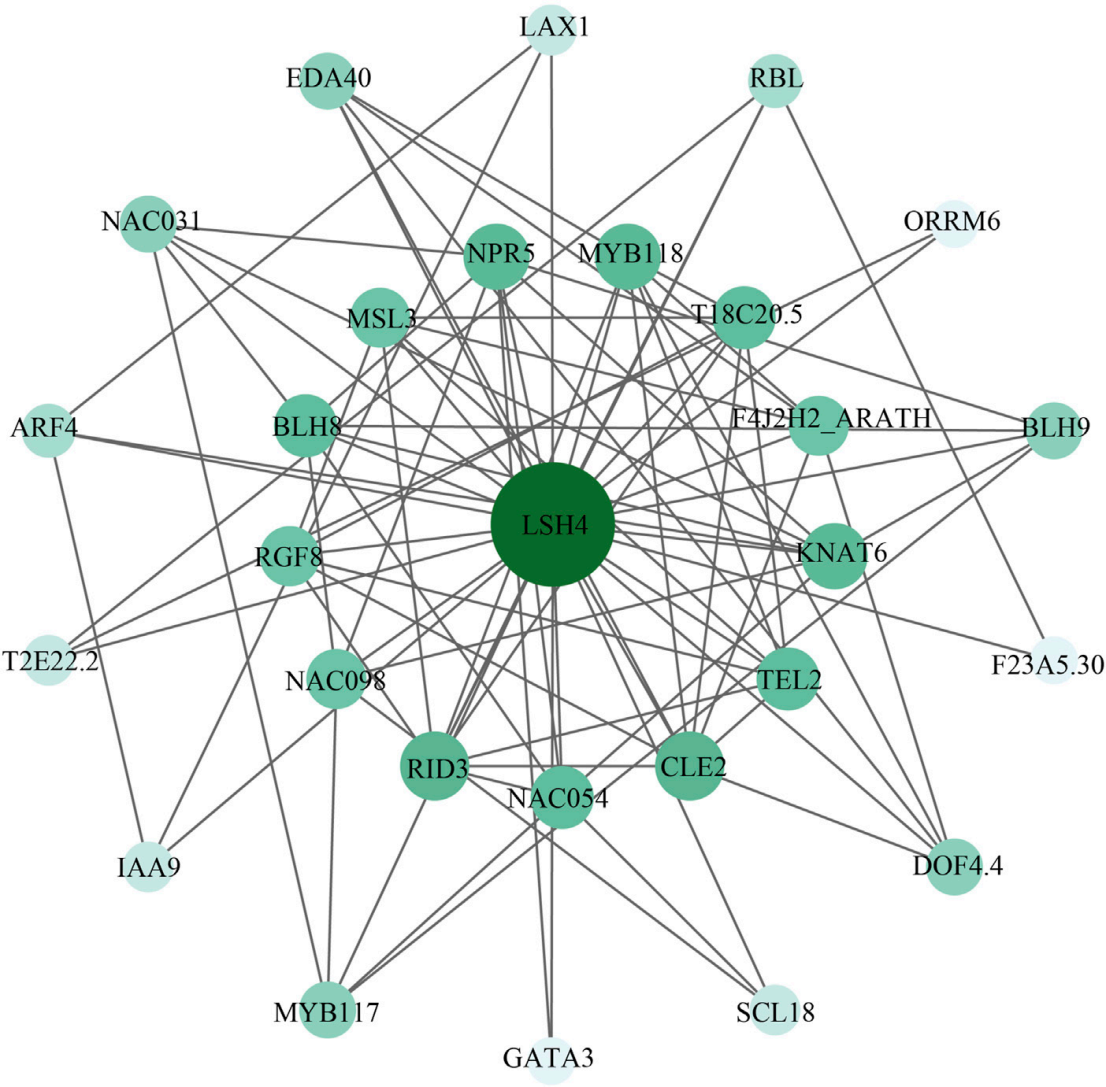
Protein interaction network of Ghir_D09G023550.1. LSH4 is the ortholog of Ghir_D09G023550.1 in *Arabidopsis*. The darker the color and the bigger the node, the higher the node degree.

First of all, an interaction network was constructed between cotton ALOG proteins, and the results showed that they do not interact with each other. Furthermore, we predict the possible regulatory mechanism of Ghir_D09G023550.1 based on LSH4 (Light-dependent Short Hypocotyls 4), the protein with the highest homology to Ghir_D09G023550.1 in Arabidopsis (Figure 7). LSH4 belongs to the ALOG family which may act as a developmental regulator by promoting cell growth in response to light and suppress organ differentiation in the boundary region. LSH4 is an important component of the Arabidopsis development network (Rieu et al., 2024), and mainly interacted with five types of biological process, including secondary shoot formation (GO:0010223), meristem development (GO:0048507), anatomical structure formation involved in morphogenesis (GO:0048646), formation of plant organ boundary (GO:0090691), and plant organ formation (GO:1905393).

The interaction study of cotton ALOG family provides important clues for further study of its function.

## Discussion

ALOG gene family plays important roles in plant growth and development (Naramoto et al., 2020). Our results have shown that ALOG proteins do not interact with other ALOG proteins, which suggests that they may independently participate in the growth and development. Furthermore, the expression of ALOG genes in specific tissues is significantly higher than that in other tissues, therefore, we speculate that the biological functions of ALOG family have differentiated in evolution, and they play their own roles in different tissues of plants. ALOG (called LSH in Arabidopsis) family in *Arabidopsis thaliana* play regulatory roles in various aspects of plant growth and development. *Arabidopsis thaliana* genome contains 10 ALOG genes that are involved in different aspects of growth and development. AtLSH1 plays a role in light regulation during seedling development, and its function relies on phytochromes. Overexpression of AtLSH1 and AtLSH2 greatly inhibited hypocotyl elongation in a light independent manner and reduced both vegetative and reproductive growth. AtLSH3 and AtLSH4 play a role in inhibiting organ differentiation at the boundary region. AtLSH8 positively regulates ABA signaling by changing the expression pattern of ABA responsive proteins. AtLSH10 potentially representing a general mechanism for the specific function of plant histone deubiquitinates at their target chromatin (Naramoto et al., 2020; Rieu et al., 2024). In this study, 42 ALOG genes were identified from allotetraploid cotton species *Gossypium barbadense*. Like ALOG genes in other species, these genes are also probably involved in different aspects of cotton growth and development. Based on the expression data, we speculate that Gbar_D05G011080, Gbar_D03G015070, Gbar_D08G022570 and Gbar_A08G025590 might be involved in cotton fiber development.

The ALOG gene family occurred before or during the plant terrestrialization process, exhibiting functional conservation and diversification during the evolution of land plants (Iyer and Aravind, 2012; Turchetto et al., 2023; Xiao et al., 2018). Genomes of land plants have experienced extensive genome-wide and regional duplications. Gene duplication expands the ancient ALOG gene family and produce multiple redundant paralogs.

The evolutionary fates of duplicated genes shape phenotypic stability and allow them to compensate each other’s loss. The allotetraploid *G. hirsutum* and *G. barbadense* originated from interspecific hybridization between the A-genome *G. arboreum* and the D-genome *G. raimondii*. In this study, we found that the sum of ALOG genes in the two diploid progenitors exceeds the number of ALOG genes in the tetraploid genome. This suggests that gene loss occurred during the polyploidization process.

Cis-elements in gene promoter regions serve to play critical roles in regulating gene expression (Wittkopp and Kalay, 2011). The results of our analysis indicated that the proportions of cis-elements across different cotton species are similar (Figure 4). The selective pressure of a gene family can be reflected by Ka/Ks ratio.

The results of our analysis also indicated that the ratio has a similar distribution pattern both within the same cotton species or between different cotton species (Figure 3). Furthermore, our molecular clock analysis indicated that the divergence time of ALOG genes took place near 15 MYA and 110 MYA which is before divergence of *Gossypium* species (Figure 3). Therefore, our findings indicate that the ALOG family was conserved during the divergence of *Gossypium* species.

Gene families originated from duplication of the same ancestor (Xu et al., 2012). Whole genome duplication, segmental duplication, tandem duplication and transposable elements provides major forces that drive the duplication of gene families (Cannon et al., 2004). In this study, only one tandemly duplicated gene pair was fond in *G. arboretum*, however, whole genome duplication, segmental duplication and transposable elements all play important roles in the duplication of ALOG gene family.

Up to now, the functions of ALOG genes are only available for Arabidopsis, rice, and tomato. This study revealed the ALOG gene family in cotton, and explored their evolution, biological function and expression profiles. In future work, we will integrate multiple methods to study the functions of each cotton ALOG gene and we are confident that this will accelerate cotton breeding.

## Conclusion

In conclusion, we identified 135 members of the cotton ALOG gene family. Except for 3 ALOG proteins that contain additional LRR domain, all other members have only an ALOG domain. The Ka/Ks ratio between orthologous gene pairs revealed that ALOG genes had undergone purifying selection during evolution. Most ALOG genes do not contain introns, and their conserved motifs, cis-elements, gene duplications, and expression patterns were analyzed. The results of this study provide a basis for the future exploration of the function of ALOG genes.

## Data Availability

The original contributions presented in the study are included in the article/Supplementary Material, further inquiries can be directed to the corresponding author.
